# The Extended Cleavage Specificity of Human Thrombin

**DOI:** 10.1371/journal.pone.0031756

**Published:** 2012-02-27

**Authors:** Maike Gallwitz, Mattias Enoksson, Michael Thorpe, Lars Hellman

**Affiliations:** Department of Cell and Molecular Biology, Uppsala University, Uppsala, Sweden; University of Washington, United States of America

## Abstract

Thrombin is one of the most extensively studied of all proteases. Its central role in the coagulation cascade as well as several other areas has been thoroughly documented. Despite this, its consensus cleavage site has never been determined in detail. Here we have determined its extended substrate recognition profile using phage-display technology. The consensus recognition sequence was identified as, P2-Pro, P1-Arg, P1′-Ser/Ala/Gly/Thr, P2′-not acidic and P3′-Arg. Our analysis also identifies an important role for a P3′-arginine in thrombin substrates lacking a P2-proline. In order to study kinetics of this cooperative or additive effect we developed a system for insertion of various pre-selected cleavable sequences in a linker region between two thioredoxin molecules. Using this system we show that mutations of P2-Pro and P3′-Arg lead to an approximate 20-fold and 14-fold reduction, respectively in the rate of cleavage. Mutating both Pro and Arg results in a drop in cleavage of 200–400 times, which highlights the importance of these two positions for maximal substrate cleavage. Interestingly, no natural substrates display the obtained consensus sequence but represent sequences that show only 1–30% of the optimal cleavage rate for thrombin. This clearly indicates that maximal cleavage, excluding the help of exosite interactions, is not always desired, which may instead cause problems with dysregulated coagulation. It is likely exosite cooperativity has a central role in determining the specificity and rate of cleavage of many of these in vivo substrates. Major effects on cleavage efficiency were also observed for residues as far away as 4 amino acids from the cleavage site. Insertion of an aspartic acid in position P4 resulted in a drop in cleavage by a factor of almost 20 times.

## Introduction

Proteases are essential for a large number of important biological processes such as fertilization, blood clotting, food digestion and immunity, where they constitute approximately 2% of the total human proteome [1]. A key to the regulation of these processes is their ability to select the correct targets among a myriad of substrates. This is made possible by the specific recognition of substrate sequences containing typically 7–8 contiguous amino acid (aa) residues [2]. Some proteases are highly specific, having relatively strict preferences for the majority of these 7–8 aa and therefore only cleave a few selected targets, whereas others cleave almost any substrate with the preferred aa in the P1 position, i.e. adjacent to where the peptide bond is cleaved. Experimental identification of the recognition sequences adds very important information about a protease's biological function, facilitates the identification of proteases for site-specific proteolysis, provides a basis for the design of good substrates for kinetic studies and helps in the design of efficient inhibitors. There is also considerable medical interest in proteases, with an estimated 14% of all human proteases being investigated as potential targets in drug development [3].

Thrombin is arguably the most extensively studied of all human proteases. It is a serine protease with essential functions in blood coagulation and in numerous other regulatory processes. Known natural substrates for thrombin include coagulation factors V, VIII, XI and XIII, protein C and fibrinogen [4]. It also activates platelets via cleavage of protease-activated receptors (PAR) -1, -3 and -4. Interestingly, thrombin regulates the coagulation process both positively, by cleaving prothrombin, FV and FVIII and negatively, by cleaving protein C (reviewed in [4], [5], [6]). Due to its vital importance, the substrate recognition profile of thrombin has been studied in detail since the early 1980s [7], [8]. Various techniques have been used, including chromogenic peptide substrates and combinatorial methods using libraries of substrate peptides with fluorogenic leaving groups or fluorescence-quenched substrates (see Table 1) [9], [10]. These studies have shown a strong preference for arginine in position P1 and for proline in position P2 [7], [8], [11], [12], [13], [14]. Aliphatic aa have been seen to be preferred in position P4 [14], [15]. Position P1′ almost always has serine, threonine, glycine or alanine [14], [16], [17]. Aromatic aa are favored in position P2′ [18], [19], and basic residues in position P3′ [18], [19], [20]. Acidic residues are avoided, especially in positions P3 and P3′ [13], [14], [17]. These studies, which are summarized in Table 1 have resulted in a relatively detailed picture of the cleavage specificity of thrombin. However, there are limitations with these studies. The preferences for aa N terminally or C terminally of the cleavage site have been determined separately. In other studies, one or several positions have been fixed or only a limited number of combinations have been tested. Interactions depending on subsite cooperativity are subsequently and easily overlooked. To overcome these problems we have now determined the extended cleavage specificity of thrombin using phage substrate display technology. This method utilizes a library of approximately 5×10^7^ bacteriophages [21] where one capsid protein displays a randomized, individual oligopeptide sequence coupled to a six histidine purification tag. Protease-susceptible oligopeptide sequences are identified and amplified, usually in five rounds of selection, so that all final sequences have been selected by the protease of interest during five different occasions. The competition of suitable targets at a low concentration with countless non-substrate molecules for access to the active site probably closely resembles *in vivo* situations.

**Table 1:** Comparison of selected studies since 1981 establishing the substrate recognition sequence of thrombin.

Study(Reference)	Method	P4	P3	P2	P1	P1′	P2′	P3′	P4′	Remarks
Pozsgay 1981 (7)	35 pNA chromogenicsubstrates	n.d.	**Bulky** **D-aa**	**P**	(R)	n.d.	n.d.	n.d.	n.d.	Subsite coopera-tivity
Lottenberg 1983 (8)	24 pNA chromogenicsubstrates	n.d.	**HydroPho-bic**	**P**	**R**	n.d.	n.d.	n.d.	n.d.	
Chang 1985 (12)	Polypeptide hormones and derivatives	**Hydrophobic**	**Hydrophobic**	**P** **G**	**R** **R**	**Not DE** **G**	**Not DE**	-	-	Natural peptides selected by homology
Chang 1985 (36)	Digestion of mouse kappa light chains	-	-	**P** **V**	**R** **K**	**T** **S**	-	-	-	to chromo-genic substrates
Kawabata 1988 (11)	Boc-XZR-NH-Med(X:12, Z:15)	n.d.	**D (O-Bzl)**	**P**	(R)	n.d.	n.d.	n.d.	n.d.	Not all aa represented
Le Bonniec 1991 (13)	1) 17 pNA chromogenic substrates	n.d.	**(n.d.)**	**P/A/GV**	**R**	n.d.	n.d.	**n.d.**	n.d.	
	2) Mutagenesis of peptides corresponding to protein C P7-P5′	(V)	**Not D**	(P)	(R)	(L)	(I)	**Not D**	(G)	Subsite coopera-tivity
Ebert 1991 (20)	Mutagenesis of R to S or N in fibrinogen Aα	(G)	(G)	(V)	(R)	(G)	(P)	**R**	(V)	Only R, S or N in P3′
Theunissen 1993 (17)	Mutagenesis of antithrombin-III (pseudosubstrate)	(I)	**(A) Not D**	(G)	(R)	**SA/G/T**	(L/V)	**(N)** **Not E**	(P)	Subsite coopera-tivity
Le Bonniec 1996 (18)	21 fluorescence-quenched substrates(Abz-VGPRSXXLK(Dnp)D)	(V)	(G)	(P)	(R)	(S)	**F/W/A** **not D/E**	**K/W/Q** **Not D**	(L)	R not among 10 repre-sented aa
Vindigni 1997 (37)	8 pNA chromogenic substrates (P1: R/K; P2: P/G; P3: V/F)	n.d.	**V**	**P**	**R**	n.d.	n.d.	n.d.	n.d.	Subsite coopera-tivity
Marque 2000 (19)	38 fluorescence-quenched substrates(Abz-VGPRSXXLK(Dnp)D)	(V)	(G)	(P)	(R)	(S)	**F/Y/W/R**	**R/K**	(L)	X is not C
Backes 2000 (15)	Fluorogenic substrate library, 6859 members(Ac-XXXK-AMC)	**NIle/LIF**	**X**	**P**	(K)	n.d.	n.d.	n.d.	n.d.	P1: K
Petrassi 2005 (14)	1) Fluorogenic positional scanning; 6 wells à 361 substrates	**NIle/L**	**Q/S/T/R**	**P**	**R**	n.d.	n.d.	n.d.	n.d.	X is not C
Petrassi 2005 (14)	2) Biased donor-quencher library; 19 sublibraries à 6859 members (LTPRXXXX)	(L)	(T)	(P)	(R)	**S/AT/G**	**Not DE**	**Not DE**	**X**	No specific P3′ and P4′ preference found
**This study**	Phage-displayed 9-mer library, ∼5×10^7^ members	**L/** ***G***	***G*** **/T/R/MV**	**P/G/V**	**R**	**SAG/T**	**W/G/FS**	**R**	**V/LSR**	Subsite coopera-tivity

Letters in bold indicates investigated positions, residues that were held constant are in parentheses. The preferred amino acids are denoted in the order of preference. Equally favorable residues are indicated by the absence of a slash (/). n.d., not determined; -, not applicable; pNA, para-nitroanilide.

Phage display allows the simultaneous investigation of primed and non-primed substrate positions, and can inform about subsite cooperativity. Compared to the analysis of individual peptides, which is also sensitive to subsite cooperativity, phage display has the advantage that numerous sequences can be investigated in a short time. Other advantages include that phage display is virtually unbiased, works independently of the P1 specificity and tolerates big variations in the degree of selectivity. It is only when proteases requiring a three-dimensional substrate structure that is not provided by phage-displayed peptides the method may fail [22].

Phage display has already been successfully applied to proteases preferring various aa in the P1 position including aspartate (Granzyme B), glutamate (ADAMTS-4), arginine (OmpT, kallikrein-2), phenylalanine (rMCP-4, mMCP-1, mMCP-4, human chymase, dog chymase), tryptophan (opossum chymase) alanine or asparagine (MMP-11), or valine/alanine/isoleucine (rMCP-5) [21], [23], [24], [25], [26], [27], [28], [29], [30], [31], [32], [33]. Consensus motifs identified from such analyses are present in physiological substrates and can be used in the search for novel targets. Peptides corresponding to the consensus motifs and mutations of these sequences can also be used for kinetic analyses.

In this communication we present a detailed analysis of the extended cleavage specificity of the active site of human thrombin, minimizing the influence on cleavage specificity by long-distance exosite interactions. This analysis conforms very well to the previously observed preferences, as summarized in Table 1. In addition, the phage display results suggest a cooperative or additive effect between subsites P2 and P3′. A comparison between the consensus sequence and a panel of known *in vivo* substrates also showed that no natural substrates display the consensus sequence but represent sequences that show only 1–30% of the optimal cleavage efficiency for thrombin. This very interesting finding indicates that maximal cleavage, in the absence of exosite interactions, is not always desired but instead may cause problems with excessive or dysregulated coagulation. A low cleavage rate of the selected sequence may be strongly enhanced by strong and specific exosite interactions.

Moreover, we present a screening of the human proteome for potential novel thrombin targets using the derived consensus cleavage motif, Pro-Arg-[AlaGlySerThr]-[not AspGlu]-Arg (i.e. P-R-[AGST]-[not DE]-R). A list of 73 such potential targets is presented where the majority are involved in cell adhesion, the nervous system, development/differentiation and circulatory homeostasis. Some of them may prove to be novel important targets for this multifaceted enzyme.

## Results

### Phage display analysis of the extended cleavage specificity of human thrombin

**Figure 1:**
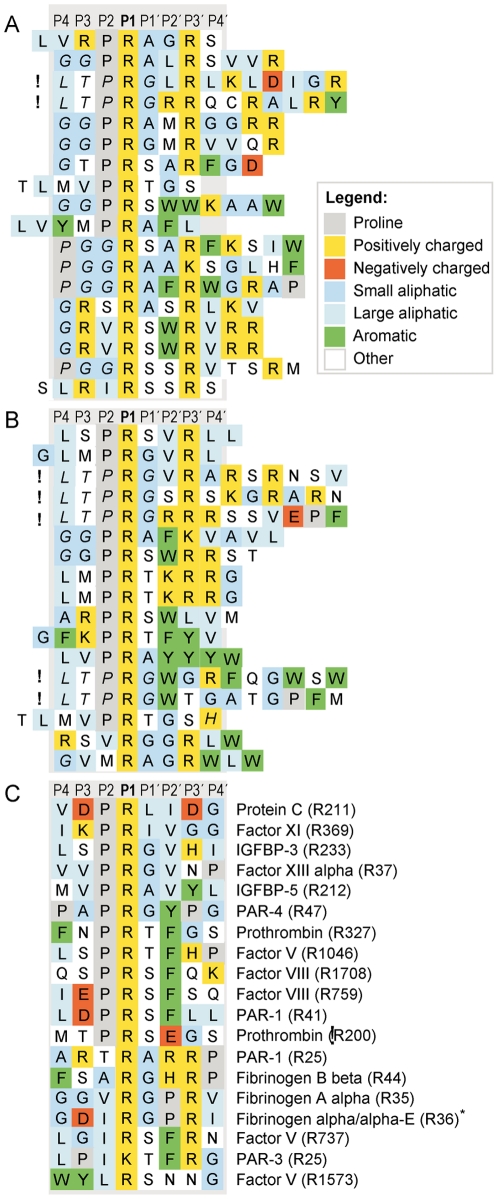
Alignment of sequences obtained after five selection rounds with 1 U of thrombin or 0.2 U of thrombin, compared to natural substrates. Panel A shows the result with 1 U of thrombin, panel B the result with 0.2 U of thrombin and panel C a panel of natural substrates. The P1 residue in natural substrates (after which cleavage occurs) is denoted in parentheses. Substrate sequences refer to *Homo sapiens* where not indicated otherwise.! marks phage sequences that have LTPRG instead of LTPGG in the N-terminal flank. Residues from the non-randomized phage region are in italics. *, from *Rattus norvegicus*; IGFBP, insulin-like growth factor-binding protein; PAR, protease-activated receptor. The cleavage site of thrombin in the natural substrates listed in panel C is numbered from the N terminal of the pre-pro protein, from the first methionine. This list of natural substrates is a selection of a few of the most well known substrates of this enzyme. However, the list of potential in vivo substrates is much longer and includes many other proteins such as protein S, TAFI, antithrombin, heparin cofactor II and nexin I.

A library of T7 phage-displayed nanomer peptides was subjected to five rounds of selection with 0.2 U or 1 U of human thrombin (1.5 and 7.5 nM of thrombin) [21], [33], [34]. This library contains approximately 5×10^7^ independent bacteriophages [21]. The ratio of phages released in thrombin-treated samples compared to the PBS control increased steadily with each selection round, reaching 240 in samples with 1 U thrombin and 136 with 0.2 U of thrombin after five selection rounds (data not shown). Seventeen or eighteen DNA sequences coding for cleavage-susceptible peptides were sampled from plaques representing selection with 0.2 U or 1 U of thrombin, respectively. All sequences were aligned to the most frequently observed pattern of at least four aa, i.e. [other]-[basic]-[small hydrophobic]-X-[basic] (Fig. 1A, 1F). The consensus could then be refined to P-R-[AGST]-[not DE]-R. This consensus closely reflects the collected results from thirteen previous studies (Table 1). The strongest preference was observed for the first arginine in the consensus, which is therefore likely to represent the P1 position as determined from the phage display results. This is in accordance with previously established data (see Table 1).

Notably, we retrieved seven inserts from thrombin-selected phages where the sequence flanking the random nonapeptide amino-termini was mutated to encode Leu-Thr-Pro-*Arg*-Gly instead of Leu-Thr-Pro-*Gly*-Gly (“!” in Fig. 1). Five of these sequences have arginine in position P3′, in accord with the refined consensus. We have never before observed mutations in the non-randomized region of selected peptides [21], [28], [33]. The retrieval of these sequences in the present study demonstrates that even very infrequent sequences that represent good substrates can be recovered by phage display.

### Amino acid prevalence in positions P4 to P4′ as derived by phage display conforms with natural substrates and previous studies

**Figure 2:**
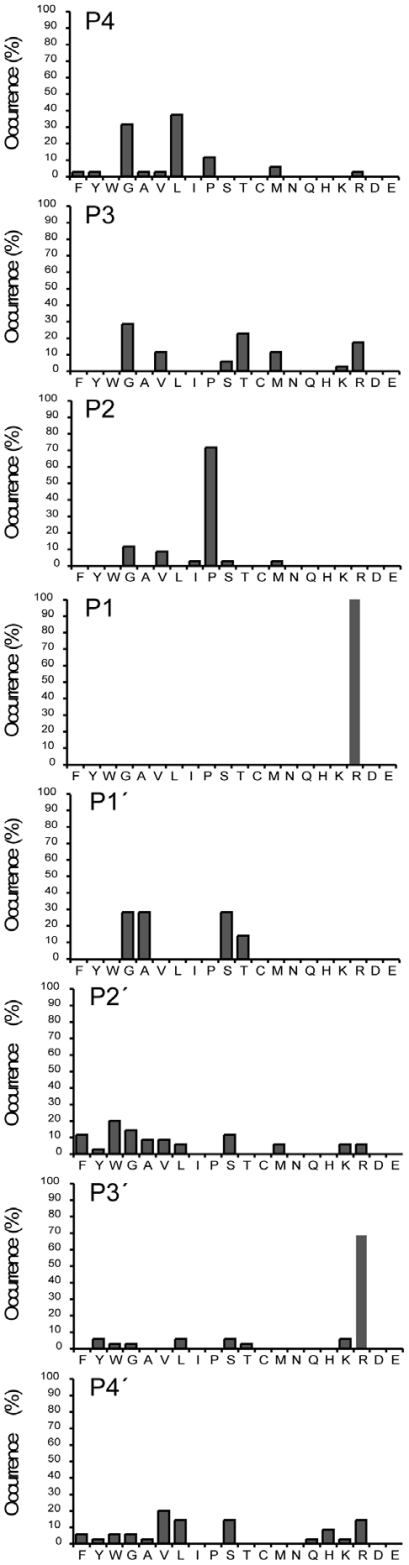
Amino acid frequency in positions P4 to P4′ of thrombin-susceptible phage sequences. This analysis is based on the alignments shown in Figure 1A and 1B. For clarity, amino acids are displayed in functional groups, starting to the left with aromatic residues, and ending with acidic residues to the right.

Based on our alignments, we analyzed the prevalence of aa in each single position (Fig. 2) and, as stated above, thrombin's long-known requirement for arginine in position P1 was reproduced [8], [9], [12], [35]. In position P2, the well-established proline [7], [8], [11], [12], [36], [37] dominated (71%), but also aliphatic aa were tolerated. P2 glycine, valine or isoleucine were together present in 23% of the sequences. Although several earlier studies report similar findings, recent studies have mostly focused on P2 proline (see Table 1). However, aliphatic P2 residues are present in a number of natural thrombin substrates (Fig. 1C), including fibrinogen Aα and Bβ, two cleavage sites in factor V (R737 and R1573), and PAR-3.

Position P3 was not very restricted, but excluded negatively charged aa. The most frequent residues here were glycine (29%), threonine (23%) and arginine (17%) (Fig. 2), three aa with differing biochemical and structural characteristics. A broad specificity as well as an exclusion of acidic residues, has previously been observed for position P3 [8], [13], [14], [15]. Intriguingly, several natural substrates have acidic P3 residues, e.g. factor VIII (site R759), PAR-1 (site R41), rat fibrinogen α/α-E and protein C. The negative contribution of the acidic residue may here be compensated for by exosite interactions. In line with this view, a synthetic peptide corresponding to protein C residues P7 to P5′ is in itself a poor thrombin substrate [13].

A more restricted preference was found in position P4, with aliphatic glycine or leucine in 31% or 37% of the sequences, respectively. This is in accordance with previous reports [14], [15]. Furthermore aliphatic P4 residues are frequently found in natural substrates (Fig. 1 and [12]).

On the primed side, in position P1′, we found only glycine (29%), alanine (29%), serine (29%) or threonine (14%). Studies using mutagenesis analysis [17] or a fluorescence-quenched library [14] have reported the same four preferred P1′ residues (see Table 1). These P1′ aa are also very common among natural substrates and active-site inhibitors of thrombin (Fig. 1 and [12], [36], [38]).

Position P2′ displayed rather broad specificity, with aromatic residues (phenylalanine, tyrosine, tryptophan) in 34%, and aliphatic residues (glycine, alanine, valine, leucine) in 37% of the sequences. Aromatic and aliphatic P2′ aa are frequent in natural substrates (Fig. 1 and [12], [36]). Preference for aromatic P2′ residues has also been reported from studies with fluorescence-quenched substrates [18], [19].

In position P3′, we observed a strong preference for arginine (69%), similar to results obtained with fluorescence-quenched substrates [18], [19]. The possibility of subsite cooperative effects involving position P3′ are discussed below.

Position P4′ was quite unspecific. The five most frequent aa were valine (20%), leucine (14%), serine (14%), arginine (14%) and histidine (9%). Aliphatic residues are frequent in the P4′ position of natural substrates, but P4′ is probably not a major specificity determinant. One previous study including the P4′ position also found a broad tolerance of aa [14].

### Phage display results indicates an arginine in position P3′ is important in substrates lacking proline in position P2

After aligning the phage-displayed peptides, we analyzed the representation of the consensus within the single sequences. Interestingly, we observed that all thrombin-susceptible peptides with residues other than proline in position P2 hold arginine/lysine in position P3′, whereas this is the case in only 64% of the peptides with a P2 proline (Fig. 1). This indicates that binding of substrate residues P2 and P3′ to their thrombin subsites may be partially interdependent (subsite cooperativity).

Natural substrates where P2 is not proline, such as fibrinogen Aα and Bβ, factor V (site R737), PAR-1 (site R25) and PAR-3 hold arginine in position P3′, whereas most substrates with proline in position P2 do not hold arginine in the P3′ position (Fig. 1C). The phage display results indicate that P2 proline and P3′ arginine are not mutually exclusive. Rather, the absence of an advantageous P2 residue, proline, in some substrates seems to be compensated for by the presence of an advantageous P3′ residue, arginine.

### Verifying the consensus sequence by the use of a new type of recombinant substrate

**Figure 3:**
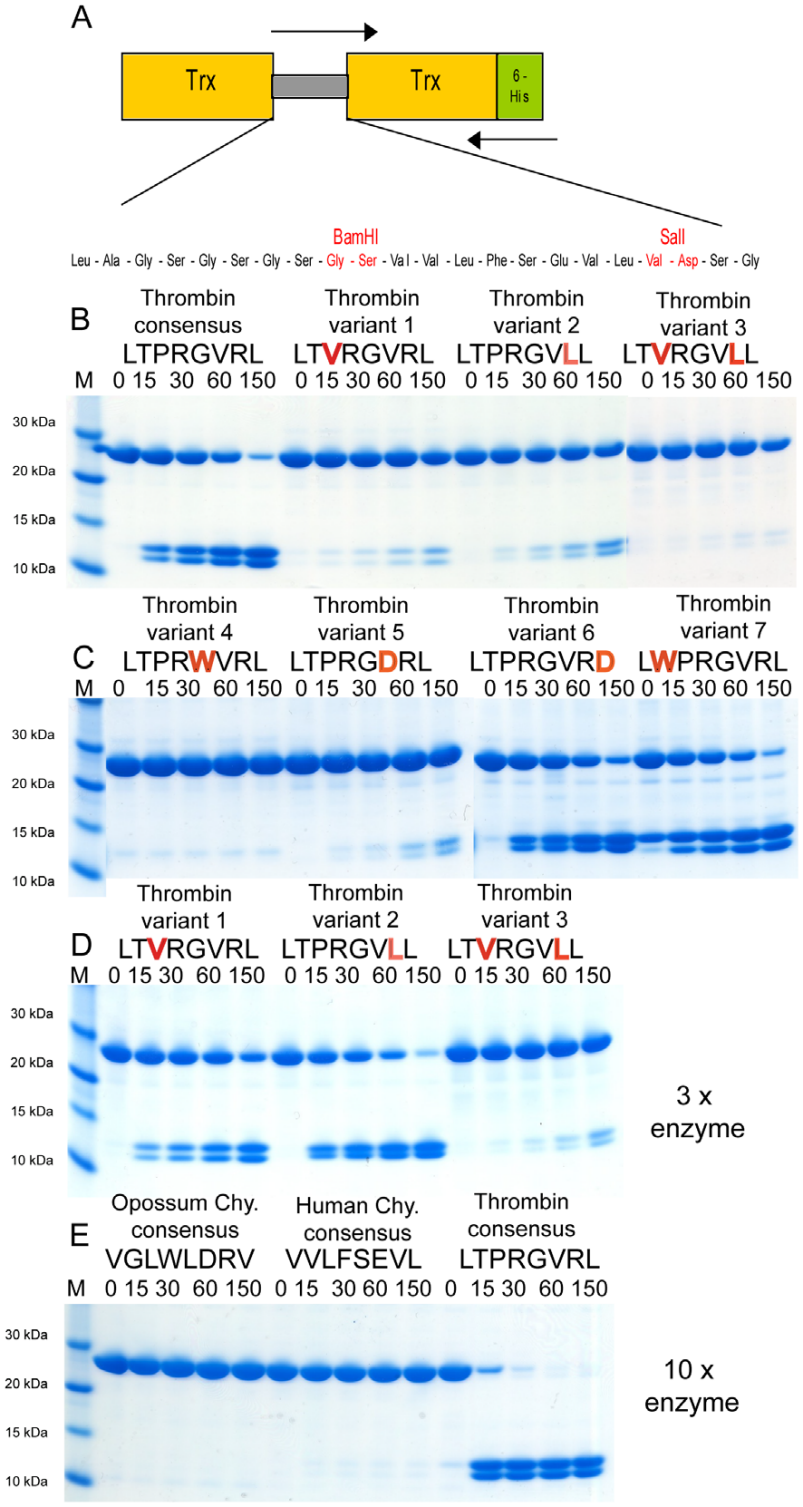
Analysis of the cleavage specificity by the use of new types of recombinant protein substrate. Panel A shows the overall structure of the recombinant protein substrates used for analysis of the efficiency in cleavage by thrombin. In these substrates two thioredoxin molecules are positioned in tandem and the proteins have a His_6_-tag positioned in their C termini. The different cleavable sequences are inserted in the linker region between the two thioredoxin molecules by the use of two unique restriction sites, one *Bam HI* and one *SalI* site, which are indicated in the bottom of panel A. Panels B to E shows the cleavage of a number of substrates by thrombin, where individual amino acids has been changed from the thrombin consensus sequence. The name and sequence of the different substrates are indicated above the pictures of the gels. The time of cleavage (in minutes) is also indicated above the corresponding lanes of the different gels. The uncleaved substrates have a molecular weight of approximately 25 kDa and the cleaved substrates appear as two closely located bands with a size of 12–13 kDa.

**Figure 4:**
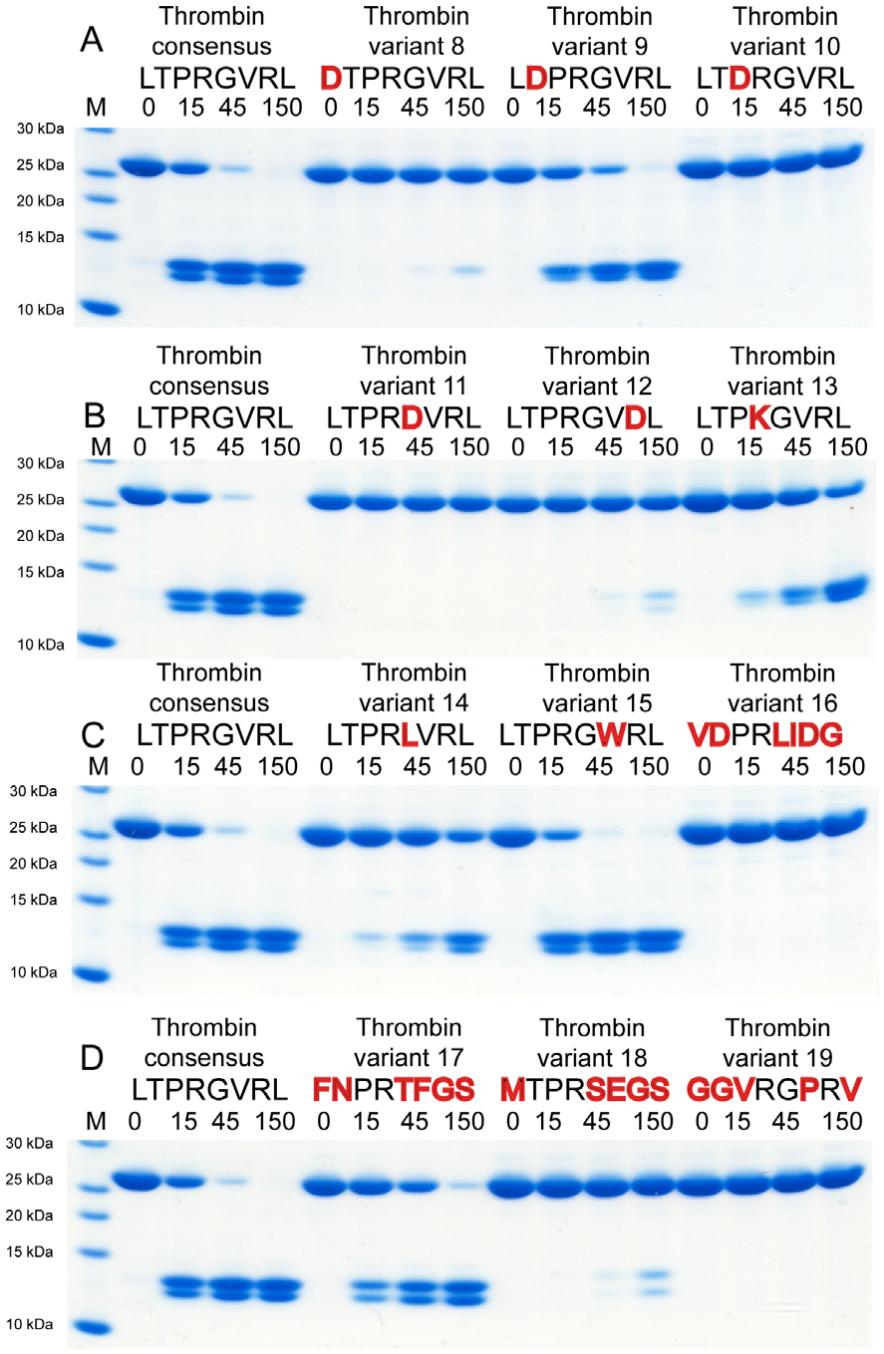
Analysis of the cleavage specificity by the use of new types of recombinant protein substrate. Panels A to D shows the cleavage of a number of substrates by thrombin, where individual amino acids has been changed from the thrombin consensus sequence. The name and sequence of the different substrates are indicated above the pictures of the gels. The time of cleavage (in minutes) is also indicated above the corresponding lanes of the different gels. Variants 16 is the Protein C cleavage site R211, variant 17 is the Prothrombin site R327, variant 18 is the Prothrombin site R200 and variant 19 is the Fibrinogen A alpha site R35 (see Figure 1).

In order to verify the results from the phage display analysis and to estimate the importance of individual aa positions for the rate of cleavage, a new type of recombinant substrate was developed. The consensus sequence obtained from the phage display analysis was inserted in a linker region between two *E.coli* thioredoxin molecules. A number of mutations in individual aa positions from this consensus sequence, the cleavage sites of a few *in vivo* substrates, and a few unrelated substrate sequences were also produced with this system. This was achieved by ligating the corresponding oligonucleotides into the *BamHI/SalI* sites of the vector (Fig. 3A and Table 2). All of these substrates were expressed as soluble proteins and purified to obtain a protein with a purity of 90–95%. These recombinant proteins were then used to study the preference of human thrombin for the different sequences (Figs. 3 and 4). The same concentration (18 µM) of all substrates was used in all experiments to obtain quantitative measurements of relative cleavage rate between the different sequences. The same concentration of thrombin (9 nM) was also used in all experiments except in two instances. When studying the cleavage of a few poor substrates for thrombin where we also determined the cleavage of the same amount of substrate, three or ten times more of the enzyme was added (Fig. 3). In most experiments the ratio substrate to protease was therefore approximately 2000.

**Table 2:** Amino acid summary of thrombin cleavage sequences.

Variant	Cleavable sequence	Variant	Cleavable sequence
Consensus	LTPRGVRL 100%	11	LTPR**D**VRL 0%
1	LT**V**RGVRL 5%	12	LTPRGV**D**L 3–4%
2	LTPRGV**L**L 8%	13	LTP**K**GVRL 15%
3	LT**V**RGV**L**L 0.3%	14	LTPR**L**VRL 15%
4	L**W**PRGVRL 100%	15	LTPRG**W**RL 150%
5	LTPR**W**VRL 0%	16	**VD**PR**LIDG** 1–2%
6	LTPRG**D**RL 5%	17	**FN**PR**TFGS** 25%
7	LTPRGVR**D** 40%	18	**MT**PR**SEGS** 1–2%
8	**D**TPRGVRL 3–4%	19	**GGV**RG**P**R**V** 1%
9	L**D**PRGVRL 5–10%		
10	LT**D**RGVRL 0%		

Amino acids shown in bold and larger font are deviations from the preferred thrombin consensus sequence. The cleavage efficiency compared to the consensus is shown as a percentage.

Thrombin was found to very efficiently cleave the consensus sequence (LTPRGVRL). By changing the proline residue in the P2 position of the thrombin consensus sequence into a valine, the second most preferred aa, based on the phage display result, (LTVRGVRL)) the efficiency of cleavage by thrombin dropped by a factor of approximately 20 (Fig. 3B and 3D). By changing the arginine residue in the P3 position of the thrombin consensus sequence into a leucine, also the second most preferred aa based on the phage display result, (LTPRGVLL)) the efficiency of cleavage by thrombin dropped by a factor of 10–15 (Fig. 3B and 3D). Altering both the proline residue in the P2 position and the arginine in position P3′of the thrombin consensus sequence into a valine and leucine respectively, (LTVRGVLL)) the efficiency of cleavage by thrombin dropped by a factor of 200–400 (Fig. 3B and 3D). These results show the major importance of these two residues in conferring the substrate specificity of thrombin.

When analyzing the phage display results in detail, we also observed that no aromatic aa are present in position P1′. This position was relatively unspecified with approximately equal representation of four different aa glycine (29%), alanine (29%), serine (29%) or threonine (14%). A tryptophan was inserted in the P1′position (LTPR**W**VRL) and tested for efficiency in cleavage. No cleavage of this substrate was observed, indicating that no large bulky aa is tolerated in this position. Similarly, in position P3 we did not observe any aromatic aa and only one example of an aromatic aa is found for this position in the natural substrates listed in Figure 1 (Factor V (R1573)). A substrate was produced where tryptophan was introduced in position P3 instead of the preferred threonine (L**W**PRGVRL). However, this mutation had no effect on the cleavage rate (Fig. 3C).

A lack of negatively charged aa in position P2′and P3′ has been observed. Therefore a mutant where an aspartic acid was inserted in position P2′ was tested (LTPRG**D**RL). This substrate showed a reduction in cleavage by approximately 15 times compared to consensus. The effect of this mutation was almost as severe as mutating the proline in position P2 and as severe as mutating arginine in the P3′position. In contrast, introducing an aspartic acid in position P4′ (LTPRGVR**D** ) had only a minor effect on the rate of cleavage, by a factor 2–3, compared to the consensus (Fig. 3C).

A number of additional substrates were also included in this study. The optimal sequence for cleavage by the human mast cell chymase (HC) and the opossum mast cell chymase (OC) have recently been determined [29], [31]. When analyzing the cleavage of these two sequences (VGLWLDRV and VVLFSEVL) we observed that human thrombin leaves these sequences completely untouched even after using 10 times more enzyme (Fig. 3E). This result shows the high selectivity in substrate selection by thrombin.

Following the initial screening we felt that these results were so interesting that we decided to extend the analysis to a number of additional substrates. From the phage display data we had observed an almost complete lack of negatively charged aa in all eight aa positions surrounding the cleavage site. Therefore an aspartic acid residue was placed in various positions in the substrate. The insertion of an aspartic acid in the P4 position ( **D**TPRGVRL) showed surprisingly a major effect on cleavage, a drop in efficacy by a factor 20–30 (Fig. 4A). Interestingly, insertion of an aspartic acid in position P3 (L**D**PRGVRL) had a much less pronounced effect. Here efficacy dropped by a factor 2–3 (Fig. 4A). However, the insertions of an aspartic acid in positions P2 or P1′ had dramatic effects (LT**D**RGVRL and LTPR**D**VRL)). An almost complete lack of cleavage was observed (Fig. 4A and 4B). Insertion of an aspartic acid in the P3′position also showed a marked effect on cleavage, by a factor of approximately 20–30 times that of the consensus sequence (Fig. 4B).

None of the substrates obtained from the phage display analysis had lysine in the P1 position. However one *in vivo* substrate, PAR-3, has been shown to have a lysine in this position (Fig. 1C). In order to test the selectivity for arginine over lysine (both positively charged aa) we exchanged arginine for lysine in one of the synthetic substrates (LTP**K**GVRL) (Fig. 4B). Interestingly the analysis of the cleavage rate of this substrate showed that arginine is preferred over lysine by a factor of approximately 10 (Fig. 4B), indicating a relatively high selectivity for arginine in the P1 position.

From the previous analysis we had seen that introduction of an aromatic aa in thee P1′ position completely blocked cleavage, therefore we decided to test other aa substitutions in this position. Introducing an aspartic acid in this position also completely blocked cleavage, whereas a leucine in this position (LTPR**L**VRL) showed an approximate 10-fold reduction in cleavage (Fig. 4C).

Insertion of a tryptophan in position P2′, instead of the consensus valine, (LTPRG**W**RL) had no or even a minor positive effect on the rate of cleavage.

When we compared the consensus sequence obtained from the phage display analysis with the list of natural *in vivo* substrates presented in Figure 1C, we observed that no *in vivo* substrate corresponded to the consensus sequence. Interestingly, the natural substrates appeared to be relatively poor substrates for human thrombin. In order to substantiate this conclusion, we selected four relatively different *in vivo* substrates and produced recombinant substrates containing the eight aa region spanning these three cleavage sites. The first substrate tested corresponded to arginine 211 in protein C (substrate number 16 in Figure 4C). This sequence ( **VD**PR**LIDG** ) was found to be a very poor substrate for thrombin. In our analysis we could not detect any cleavage after 150 minutes, which shows that it is 1% or below of the cleavage of the consensus substrate. The second *in vivo* substrate that was analyzed was the region of arginine 327 in prothrombin ( **FN**PR**TFGS** ). This substrate showed relatively good cleavage (Fig. 4D). However, only 25–30% cleavage compared to the consensus substrate was observed (Fig. 4D). The third *in vivo* substrate (substrate 18) was another cleavage site within prothrombin. This site, which corresponds to the region around arginine 200 ( **MT**PR**SEGS** ) was a poor site for thrombin cleavage. This site was 20–30 times less efficient than the consensus site (Fig. 4D). The fourth *in vivo* site that was studied was the region corresponding to arginine 35 in fibrinogen A alpha ( **GGV**RG**P**R**V** ). This site was also a very poor site for thrombin. Similarly to the protein C substrate no cleavage could be detected even after 150 minutes, again showing it is 1% or less than the activity of the consensus site (Fig. 4D). All these four “*in vivo*” substrates were cleaved at less than 25–30% efficiency compared to the consensus substrate (Fig. 4C and 4D). This confirmed our conclusion based on the phage display analysis that most natural *in vivo* substrates are relatively poor substrates for human thrombin when presented as linear peptides. Long range exosite cooperative effects here may help in increasing the local concentration of the substrate and thereby increasing the rate of cleavage. The data from the recombinant substrate analysis has been summarized in Table 2.

### Novel candidate substrates for thrombin identified by PROSITE search

Known natural thrombin substrates mostly align to only three or four positions in the consensus recognition sequence, P-R-[AGST]-[not DE]-R. Thus, database searches with the full consensus may identify novel potential thrombin substrates. We searched the Swiss-Prot, TrEMBL and PDB databases for human (H. sapiens) proteins holding the P-R-[AGST]-[not DE]-R motif, yielding 651 hits in 602 protein sequences. In at least 75 proteins, the motif was extra-cellular or secreted (Tables 3, 4, 5 and 6) and therefore potentially accessible for thrombin. Interestingly, a total 73 of these proteins seem involved in one or several of four areas including, cell adhesion, the nervous system, development and differentiation or circulatory homeostasis (Tables 3, 4, 5, and 6). More specifically, 36 proteins (48%) have been implicated in cell adhesion. Among these are eight collagen variants and integrin αV, i.e. central components of the extra-cellular matrix (ECM). Thirty-three proteins (44%) have roles in the nervous system, including three roundabout homologs and persephin, all of which are implied in neurotropic activity. Thirty proteins (40%) are involved in development/differentiation, and eleven (14.7%) in circulatory homeostasis.

**Table 3:** Potential novel thrombin substrates.

ID/Functional grouping	Name	(Presumable) function	Motif	Positions	Location in protein	Protein expression
P11230A **B** C D	Acetylcholin receptor subunit β	Synaptic transmission	PRGGR	224–228	24–244 ED (multi-pass)	Neurons, muscle
Q6UY14A B C **D**	ADAMTS-like protein 4	Stimulates apoptosis	PRGIR	424–428	Secreted	Lung, plasma, placenta,
Q9UKB5**A** B C D	Adherens junction-associated protein 1	Cell adhesion and migration	PRARR	96–100	1–282 ED	Uterus, pancreas
O00253A **B** C D	Agouti-related protein	Weight homeostasis	PRSSR	81–85	Secreted	Brain, testis lung, kidney
Q9BXJ7A B C **D**	Amnionless protein	Vit. B_12_ absorption, directs trunk mesoderm	PRSSR	195–299	20–357 ED	Kidney, testis, thymus, PBL, colon, small intestine
Q9Y5L1**A** B **C** D	Angiopoietin-related protein 3	Cell-matrix adhesion, lipid metabolism, angiogenesis	PRAPR	220–224	Secreted	Liver (kidney)
Q6SPF0A B **C** D	Atherin	Atherogenesis by immobilizing LDL in arterial wall	PRAPR	112–116	Cytoplasmic/secreted	Atherosclero-tic lesions
P01160A B **C**D	Atrial natriuretic factor	Cardiovascular homeostasis	PRSLR	122–126	Secreted; 56–122 propeptide	
O14514**A B C** D	Brain-specific angiogenesis inhibitor 1	Inhibits angiogenesis in brain; cell adhesion, signal transduction	PRSLR	861–865	31–948 ED(7-TM)	Brain
Q9NYQ6**A B** C **D**	Cadherin EGF LAG seven-pass G-type receptor 1	Cell-cell signalling during formation of the nervous system, planar polarity	PRAPR	58–62	22–2469 ED	
Q9NYQ7**A B** C **D**	Cadherin EGF LAG seven-pass G-type receptor 3	Cell-cell signalling during formation of the nervous system	PRTARPRGAR	249–2532337–2341	33–2540 ED	
Q96IY4A B **C** D	Carboxypeptidase B2 (Thrombin-activable fibrinolysis inhibitor)	Cleaves kinins and anaphylatoxins	PRTSR	33–37	Secreted; 23–114 activation peptide	Plasma; synthesized in liver
Q16619A B **C** D	Cardiotrophin-1	Induces cardiac myocyte hypertrophy	PRAPR	113–117	Secreted	Heart, ovary, prostate, skeletal muscle
Q9H2X0A B C **D**	Chordin	Key developmental dorsalizing factor	PRGCR	869–873	Secreted	Early vertebrate tissues
P02452**A B** C **D**	Collagen α1(I) chain	Epidermis and skeletal development	PRGPR	119–123	Secreted	Brain, spleen, tendon, ligaments, bones
P02461**A** B C D	Collagen α1(III) chain (Goodpasture antigen)	Homotrimers in most soft connective tissues	PRGNR	1165–1169	Secreted	Skin, placenta, liver
P20908**A** B C D	Collagen α1(V) chain	Binds DNA, heparan sulfate, heparin, thrombospondin, insulin	PRGQR	908–912	Secreted	Nearly ubiquitous
P12107**A** B C **D**	Collagen α1(XI) chain	Trimer α1(XI), α2(XI), α3(XI) may control growth of collagen II fibrils	PRGQRPRGSR	878–882887–891	Secreted	Cartilage, placenta

Hits holding the consensus P-R-[AGST]-[not DE]-R in an extra-cellular or secreted part are listed alphabetically by name. In the column to the left, **A** in bold and larger font indicates (presumable) involvement in cell adhesion, **B** in the nervous system, **C** in the cardiovascular system, and **D** in development/differentiation. ED, extra-cellular domain; 7-TM, seven-transmembrane receptor; PBL, peripheral blood leukocytes.

**Table 4:** Potential novel thrombin substrates.

ID/Functional grouping	Name	(Presumable) function	Motif	Positions	Location in protein	Protein expression
P12110**A** B C D	Collagen α2(VI) chain	Cell-binding protein	PRGPR	568–572	Secreted	Fibroblasts, placenta, uterus
P113942**A** B C **D**	Collagen α2(XI) chain	Trimer α1(XI), α2(XI), α3(XI) may control growth of collagen II fibrils	PRSARPRGQR	176–180845–849	Secreted	Cartilage
P12111**A** B C D	Collagen α3(VI) chain	Cell-binding protein	PRGNR	2366–2370	Secreted	Fibroblasts, placenta, plasma
Q14050**A** B C D	Collagen α3(IX) chain	Structural component of hyaline cartilage	PRGLR	230–234	Secreted	Skin, cartilage
Q9UQ03A **B** C **D**	Coronin-2B	Reorganization of neuronal actin structure	PRAAR	397–401		Brain
Q14118**A** B C D	Dystroglycan	Cell-matrix interaction; laminin receptor; target for M. leprae	PRTPR	453–57	Secreted;30–653 α-dystroglycan	Skeletal muscle
P27539A **B** C **D**	Embryonic growth/differentiation factor 1	Embryonic tissue differentiation	PRSLR	210–214	Secreted; 30–253 propeptide	Brain
P05305A B **C** D	Endothelin-1	Vasoconstriction	PRSKR	88–92	Secreted;90 end of big endothelin 1	Lung, placenta
Q06828**A** B C D	Fibromodulin	Rate of fibrils formation, binds to collagen type I and II	PRSLR	175–179	Secreted;168–188LRR-5 2	
P41439A B C **D**	Folate receptor γ	Binds folate	PRSAR	22–26	Secreted,1–23 signal sequence	Spleen, thymus, bone marrow; ovary and uterine carcinoma
P09681A B C D	Gastric inhibiory polypeptide	Stimulates insulin release, inhibits gastric acid secretion	PRGPR	47–51	Secreted;22–50 propetide	
O60391A **B** C D	Glutamate (NMDA receptor subunit 3B	Ion channel in prosynaptic membrane	PRALR	469–473	23–564 ED	Brain, motorneurons
Q9NZ20A B C D	Group 3 secretory phospholipase A2	Phospholipid metabolism	PRAIR	444–448	Secreted	Kidney, heart, liver, skeletal muscle
Q96RW7**A** B C D	Hemicentin-1 (Fibulin-6)	Part of ECM	PRGYR	5301–5305	5272–5307 EGF-like 5; Ca^2+^-binding	Fibroblasts, retinal pigment epithelium
Q86UW8**A B** C **D**	Hyaluron and proteoglycan link protein 4	Binds to hyaluronic acid, formation of ECM	PRGGR	169–173	Secreted;163–268 link 1	Brain
Q9UMF0**A B** C D	ICAM-5	Binds to LFA-1	PRAPR	629–633	32–825 ED	Brain
Q969P0A **B** C **D**	Immunoglobulin superfamily member 8	Cell motility and proliferation, NS development, fertilization	PRSHR	524–528	28–579 ED; 431–560 Ig-like C2 type 4	Brain, kidney, testis, liver, placenta
Q13349**A** B **C** D	Integrin αD (CD11d)	Receptor for ICAM-3 and VCAM1, lipoprotein clearance, antigen clearance	PRGQR	497–501	18–1100 ED	Blood cells
P38570**A** B C D	Integrin αE pre-cursor		PRTKR	58–62	19–1124 ED	Intraepithelial T cells
P20701**A** B C D	Integrin αL (CD11a)	αL/β2 is receptor for ICAM-1, -2, -3 and –4; cytotoxicity	PRAGR	39–43	26–1090 ED	Leukocytes
P06756**A** B **C** D	Integrin αV(CD51)	Receptor for vitronectin, fibronectin, fibrinogen, prothrombin, laminin, thrombospondin	PRAAR	274–278	31–992 ED	

Hits holding the consensus P-R-[AGST]-[not DE]-R in an extra-cellular or secreted part are listed alphabetically by name. In the column to the left, **A** in bold and larger font indicates (presumable) involvement in cell adhesion, **B** in the nervous system, **C** in the cardiovascular system, and **D** in development/differentiation. ED, extra-cellular domain; 7-TM, seven-transmembrane receptor; PBL, peripheral blood leukocytes.

**Table 5:** Potential novel thrombin substrates.

ID/Functional grouping	Name	(Presumable) function	Motif	Positions	Location in protein	Protein expression
P20702**A** B **C** D	Integrin αX (CD11c)	Receptor for fibrinogen	PRGWR	498–502	20–1107 ED	Monocytes, granulocytes
P16144**A** B C D	Integrin β4 precursor (CD104)	α6/β4 is receptor for laminin	PRGLR	378–382	28–710 ED	Epithelia
Q9Y6N6**A** B C **D**	Laminin subunit γ3	Organization of embryonic cells into tissues	PRSGR	443–447	Secreted; 430–479EGF-like 4	Skin, heart, reproductive tracts, lung
O15230**A** B C **D**	Laminin subunit α5	Organization of embryonic cells into tissues	PRSSR	3372–3376	Secreted; 3340–3513 laminin-like 4	Heart, muscle, lung, placenta, kidney, retina, pancreas
O75610A B C **D**	Left-right determination factor 1	Left-right axis determination	PRSAR	138–142	Secreted; 132–135 R-X-X-R site	Colon, pancreas, spleen
Q9NT99A B C D	Leucin-rich repeat-containing protein 4B		PRSSR	543–547	36–576 ED	
Q9NZU1**A B** C D	Leucin-rich repeat transmembrane protein FLRT1	Cell adhesion, receptor signaling	PRSLR	98–102	21–524 ED78–98 LRR299–121 LRR3	Kidney, brain
Q9NZR2A **B** C D	Low-density lipoprotein receptor-related protein 1B	Receptor-mediated endocytosis	PRSAR	2605–2609	ED 25–4444; 2590–2626 LDL receptor class A13	Thyroid and salivary gland; adult and fetal brain
Q9NPA2**A** B C D	Matrix metalloproteinase-25	Activate progelatinase 1	PRAPR	515–519	Mature form 108–539	Leukocytes, lung, spleen
P58417A **B** C D	Neurexophilin-1	Resembles neuropeptides	PRAKR	102–106	Secreted; 98–176. region III	Brain
Q9UM47A B C **D**	Neurogenic locus notch homolog protein 3 (Notch 3)	Regulates cell-fate determination	PRGFRPRGPRPRARR	109–1131308–13121567–1571	ED 40–1643 78–118 EGF-like 21289–1325 EGF-like 33	ubiquitous
Q99466A B **C D**	Neurogenic locus notch homolog protein 4 (Notch 4)	Regulates cell-fate determination. branching in the vascular system	PRGRR	1911–1915	1432–2003 extracellular. truncation	Heart, lung, placenta
Q8N729A **B** C **D**	Neuropeptide W	Regulates neuroendocrine signaling, stimulates water and food intake	PRSPR	115–119	Secreted	Substantia nigra, fetal kidney and trachea
Q14112**A** B C D	Nidogen-2	Cell-ECM interactions; binds to collagens I and IV, perlecan, laminin 1	PRSAR	145–49	Secreted;198–273 NIDO domain	Heart, placenta, bone
Q8NG85A **B** C D	Olfactory receptor 2L3	Putative odorant receptor	PRSLR	261–265	259–271 ED	
Q8NG80A **B** C D	Olfactory receptor 2L5	Putative odorant receptor	PRSLR	261–265	259–271 ED	
Q8NGY9	Olfactory receptor 2L8	Putative odorant receptor	PRSLR	261–265	259–271 ED	
A **B** C D						
O60542A **B** C **D**	Persephin	Neurotropic activity, development of the CNS	PRGAR	98–102	Secreted	
Q8TCZ9A B C **D**	Polycystic kidney and hepatic disease 1	Differentiation of bile collecting duct, biliary differentiation	PRGGR	3236–3240	24–3858 ED	Kidney, liver, pancreas
Q9P2E7**A B** C D	Protocadherin-10	Ca^2+^-dependent cell adhesion	PRTGR	395–399	19–715 ED; 251–358 cadherin 3	Brain. testis, ovary
Q9H158**A B** C D	Protocadherin alpha C1	Ca^2+^-dependent cell adhesion; specific neuronal connections in the brain	PRSAR	571–575	19–683 ED; 570–667 cadherin 6	Brain
O95206**A B** C D	Protocadherin-8	Ca^2+^-dependent cell adhesion	PRSGR	301–305	30–749 ED; 247–354 cadherin 3	Brain

Hits holding the consensus P-R-[AGST]-[not DE]-R in an extra-cellular or secreted part are listed alphabetically by name. In the column to the left, **A** in bold and larger font indicates (presumable) involvement in cell adhesion, **B** in the nervous system, **C** in the cardiovascular system, and **D** in development/differentiation. ED, extra-cellular domain; 7-TM, seven-transmembrane receptor; PBL, peripheral blood leukocytes.

**Table 6:** Potential novel thrombin substrates.

ID/Functional grouping	Name	(Presumable) function	Motif	Positions	Location in protein	Protein expression
Q9Y6N7A **B** C **D**	Roundabout homolog 1 (H-Robo-1)	Guides cellular migration, axon development	PRSHR	115–19	26–897 ED;68–164 Ig-like C2-type 1	Widely expressed (not in kidney)
Q9HCK4A **B** C **D**	Roundabout homolog 2 (ROBO2)	Guides cellular migration, axon development	PRSHR	78–82	22–859 ED;31–127 Ig-like C2 type 1	Ovary, brain
Q96MS0A **B** C **D**	Roundabout homolog 3 (ROBO3)	Guides cellular migration, axon development, spinal chord development	PRAHR	115–119	21–891 ED;64–160 Ig-like C2 type 1	
P09683A B C D	Secretin	Stimulates secretion of NaHCO_3_ from pancreas, inhibits release of gastric acid	PRARR	23–27	Secreted;19–26 propeptde	
O94933A **B** C **D**	SLIT and NTRK-like protein 3	Suppresses neurite outgrowth	PRTPR	339–343	29–654 ED	Cerebral cortex
O60721A **B** C D	Sodium/potassium/cal-cium exchanger 1	Sensory transduction, vision	PRGRR	192–196	1–452 ED	Retinal rod
Q99523A **B** C **D**	Sortilin	Clearance receptor on cell surface, promotes neuronal apoptosis, receptor for neurotensin, osteogenesis	PRGGR	70–74	34–77 propeptide	Brain, spinal chord, heart, thyroid, testis, placenta, skeletal muscle
A2VEC9**A B** C **D**	SCO-spondin	Formation of the CNS, neuronal aggregation	PRGWR	1579–1583	Secreted; 1564–1600 LDL-receptor class A5	
P22105**A** B C **D**	Tenascin X	Cell-ECM interactions	PRAVR	1500–1504	Secreted; 1459–1540fibronectin type II7	Fetal adrenal, testis, muscle
O95407A **B** C **D**	Tumor necrosis factor receptor super family member 6B	Inhibits apoptosis	PRAGR	246–250	Secreted	Fetal brain, lung, liver, adult tissues
Q8WY21A **B** C **D**	VPS10 domain-containing receptor SorCS1	Neuropeptide receptor	PRTPR	797–801	ED 34–1099	Fetal/infant brain, fetal retina
Q96PQ0A **B** C D	VPS10 domain-containing receptor SorCS2	Neuropeptide receptor	PRGVR	419–423	ED 51–1878	Brain, kidney
Q9Y493**A** B C D	Zonadhesin	Binding of sperm to egg	PRGLR	2622–2626	ED 18–2757	Sperms
Q9BS86**A** B C D	Zona pellucida-binding protein 1	Gamete interaction	PRAFR	50–54	Secreted (45–351)	Testis

Hits holding the consensus P-R-[AGST]-[not DE]-R in an extra-cellular or secreted part are listed alphabetically by name. In the column to the left, **A** in bold and larger font indicates (presumable) involvement in cell adhesion, **B** in the nervous system, **C** in the cardiovascular system, and **D** in development/differentiation. ED, extra-cellular domain; 7-TM, seven-transmembrane receptor; PBL, peripheral blood leukocytes.

## Discussion

Substrate phage display technology has made it possible to elucidate the substrate recognition profile of human thrombin from position P4 to P4′, completely and simultaneously. Compared to previous studies the profile obtained increases the detail substantially but also conforms relatively well with results presented in reports during the past 25 years (see Table 1). It should also be pointed out that only two previous studies report results for eight positions [14], but P1 to P4 and P1′ to P4′ were, in one of these studies, investigated separately and with two different approaches. Moreover, distinct subsites were held constant or not investigated, which effectively prevents the analysis of subsite cooperativity effects. The results for positions P2′, P3′and P4′ reported from that work were also less specific than with phage display. The second study is a phage display analysis that was performed on human thrombin and on thrombin in combination with thrombomodulin or hirugen [39]. However, only a diagram on residue preferences was included; there are no original data about individual clones. Furthermore, the high variability in the analysis made it difficult to draw any conclusions for a potential consensus cleavage site [39].

When we compare our phage display data with previous investigations the strong preferences for arginine in position P1 [8], [9], [12], [35] and for proline in position P2 [7], [8], [11], [12], [36] was reproduced. Position P3 was found to be rather unspecific. However, negatively charged aa have previously been reported not to be tolerated in this position [13]. In contrast to this finding we observe a remarkable high tolerance for most aa acids in this position including negatively charged aa (Fig. 1 and Fig. 4). Position P4 featured preferentially hydrophobic aa, namely leucine, which has been the most frequent aa reported by several studies as well as ours [14], [15]. Here we can report a remarkably high degree of specificity. Negatively charged aa are apparently not tolerated in this position as we see a drop in cleavage by 20–30 times by introducing an aspartic acid in this position (Fig. 4). For position P1′, our study and two others [14], [17] have consistently identified serine, alanine, glycine and threonine to be by far the most preferred residues. Aromatic aa were the most preferred in position P2′. Le Bonniec and Marque with coworkers have reported phenylalanine to be most preferred [18], [19] and we indicate tryptophan is the most prevalent with phenylalanine as the third most frequent aa. In position P3′, our data along with others, found arginine to be most preferred [19], [20]. Position P4′ has previously not been extensively investigated, but seems to display a preference for aliphatic residues.

Substrates aligning to the consensus in positions P1, P1′and P2′ may obtain sufficient affinity to thrombin by either a proline in the P2 position (which is well-documented), but also in the absence of P2 proline by an arginine in position P3′. However, the thrombin consensus recognition sequence does not indicate this, because most experimental thrombin substrates obtained by phage display hold both proline in position P2 and arginine in P3′. The cooperativity effects involving position P3′ have previously also been indicated from mutagenesis studies of single peptides [13], [17]. The second position involved in that study was P3, but position P2 was held constant in these studies, and several cooperativity mechanisms may exist.

Several important physiological thrombin targets do indeed lack proline in position P2 and hold arginine in P3′, e.g. fibrinogen chains Aα and Bβ, factor V, PAR-1 and PAR-3 (Fig. 1C). However, the preferred and activating PAR-1 cleavage by thrombin is LDPR-SFLL holding P2 proline. The P3′ arginine in fibrinogen Aα has great biologic significance, as illustrated by the fact that replacement of this residue with glycine, serine or asparagine leads to bleeding disorders [20]. This underlines that knowledge of subsite cooperativity effects can be medically very important. Subsite cooperativity is difficult to study experimentally but probably exists in many enzymes. A review on subsite cooperative effects in proteases, that summarizes the available information concerning this interesting phenomena, has recently been published [39]. A detailed study on the cleavage specificity of factor Xa by Bianchini et al from 2002 also comes to the conclusion that the efficiency in cleavage by factor Xa is primarily a result of exosite interactions and not the specificity of the active site [40]. This article also contains a detailed study of the cleavage specificity of human thrombin. In this article thrombin was used as a reference compound for the analysis of Factor Xa. By using a large panel of fluorescence-quenched substrates they mapped the cleavage specificity of thrombin between P3 and P3′residues [40]. Their results are very similar to results we obtain for this region by phage display. For example, they did see that the P3 position is relatively unspecific with a slight preference for methionine, threonine and arginine. In the P2 position they see that proline is the most preferred aa by almost one order of magnitude higher activity of this substrate than with leucine or valine in this position. In the P1′position they identify serine, alanine, glycine and threonine as the four most preferred aa. This is in full agreement with our data and with data from several other labs. However, in contrast to our results they find that phenylalanine is also well tolerated in this position [40]. In the P2′position they observe a preference for aromatic aa, which is in agreement with our results. The strong preference for arginine in the P3′position is also identical between the two studies [40].

Interestingly none of the *in vivo* substrates identified (listed in Figure 1) have both the P2 proline and the P3′arginine of the consensus site. This may indicate that suboptimal cleavage sites are preferred over the consensus site. The sequences of the *in vivo* substrates show a cleavage that is 10–100 times less efficient than the consensus site. The analysis of four selected “*in vivo*” sites also substantiate this conclusion by showing that these four sites were only cleaved at an efficacy of 1–30% of the consensus site (Fig. 4C and 4D). This is a finding that may seem puzzling. However, a too efficient cleavage may potentially cause excessive coagulation and a risk of unwanted thrombosis. A similar situation has been observed in the skin where the serine proteases kallikrein 5 and 7 are present in a region where the pH is suboptimal for maximal cleavage [41]. Both of these enzymes have a slightly basic pH optimum, whereas the outer layer of the skin has an acidic pH. By artificially increasing the pH, as for example by the use of neutral or alkaline soaps, the serine protease activity increases [42]. This increase in protease activity leads to premature degradation of corneodesmosomes, inactivation of β-glucocerebroside and acidic sphingomyelinase and subsequent impairment of epidermal barrier function [41], [42]. Here the protease activity has to apparently be kept under suboptimal conditions as to not cause tissue damage.

A second and more likely explanation is that long distance subsite cooperative effects play a major role in determining the specificity [43], [44]. It is well known that the specificity of the interaction between thrombin and its substrates stems not only from the interaction of the substrate with the catalytic subsites from S4 to S4′, but also from interactions with the anion binding exosites I and II, also called the fibrinogen-recognizing exosite and the heparin-binding exosite, respectively. These two exosites are positively charged domains that flank the active site. These sites interact with negatively charged regions of the substrate. Such interactions may thereby compensate for lack of direct strong interactions with the active site. There is even indications that exosite interactions may be the major source of substrate specificity for some targets [44]. The cleavage of protein C by thrombin does, for example, increase by approximately 1500 fold by interaction with thrombomodulin and by approximately 10 000 fold by interaction with thrombomodulin in the presence of phospholoipid membranes [43]. The mechanisms of this cooperative effect is not fully known but it is unlikely that the exosite interaction has any dramatic effect on the specificity of the active site of thrombin. Instead, the effect of the exosite is probably primarily in recruiting the substrates. A potent such recruitment effect may result in an increase in the local concentration of the substrate and a more efficient cleavage.

The list of potential natural substrates for thrombin in Figure 1 is by no means a complete list. Many other potential in vivo sites have been identified. For example, three sites identified in the thrombin sensitive region of protein S (R49, R60 and R70) also have sequences that indicate that they are far from optimal sites for thrombin (VCL**R**SFGT, TAA**R**QSTN, PDL**R**SCVN) [45], One important cleavage site for thrombin in thrombin-activable fibrinolysis inhibitor (TAFI) is arginine 302 [46]. This site (SYT**R**SKSK) also markedly differs from the consensus site. It thereby appears as if the absolute majority of the identified in vivo sites for thrombin are relatively poor sites for this enzyme and that other interactions probably play a major role in determining the efficiency in cleavage.

The availability of the substrate site also probably has a major impact on the efficiency of cleavage. If the site is exposed, in an accessible surface loop, it may be efficiently cleaved. However, if the site is located in a region where thrombin has difficulties in contacting the site, the cleavage may be very inefficient irrespective if the consensus sequence is present. The recombinant substrates used in this analysis have the cleavable sequences in an accessible conformation, which enables an unbiased comparison of the cleavage specificity, whereas the natural substrates may vary considerable in accessibility. This has to be taken into consideration when comparing the efficiency in cleavage of the different natural substrates. However, the consensus cleavage site for thrombin is relatively heavily charged with two arginines as well as having a proline, which introduces a bend in the peptide chain indicating that most consensus sites are probably exposed on the surface of the potential target molecules.

The phage display analysis also resulted in several additional findings concerning important restrictions in aa tolerated in various positions of the cleavage site for human thrombin. For example, the complete lack of aromatic aa in the P1′position of the substrates was very interesting. The aa in this position seems to be of major importance as very little variation is observed among all identified *in vivo* substrates and no aa other than glycine/alanine/serine and threonine was seen among the substrates obtained in the phage display analysis (Fig. 1). The only *in vivo* sites for thrombin that avoid this pattern are protein C and coagulation factor XI, which in this position have a leucine or an isoleucine, respectively (Fig. 1). Introduction of a tryptophan in this position of the consensus sequence resulted in a complete block in cleavage, which shows the importance of this position in substrate selection. Introducing an aspartic acid in this position also completely block cleavage whereas a leucine resulted in a reduction in cleavage rate by a factor 10 (Fig. 4C). Large aromatic aa are apparently not tolerated, possibly except phenylalanine [40], and negatively charged aa also severely effect cleavage in this position. However, aromatic aa are tolerated, and even potentially favored, in other positions, such as in the P2′ position (Figs. 1 and 4C).

Among the sequences originating from the phage display analysis we see an almost complete lack of negatively charged aa in all eight positions from P4 to P4′ (Fig. 1). Introduction of an aspartic acid into position P2 and P1′ resulted in complete block in cleavage and in the P2′position aspartic acid resulted in a drop in cleavage rate by approximately 15 times. Interestingly, the introduction of this negatively charged aa in the P4 position, which is relatively far from the actual cleavage site, resulted also in a dramatic drop in cleavage rate by a factor 20–30 (Fig. 4A). These findings indicates that introduction of a negatively charged aa in almost any position, maybe except the P4′ and the P3 positions, are accompanied with severe effects on the cleavage rate. The marked negative effect of introduction of an aspartic acid in the P4 position of substrates also shows that positions relatively far from the cleavage site can be of major importance for efficient cleavage. The kinetic parameters of an enzyme that has been established using short synthetic substrates, where the substrates normally are only three or four aa long and either lack all aa N or C terminal of the cleavage site should be considered only qualitatively. Such kinetic parameters have probably little relevance to the actual *in vivo* situation with natural protein substrates.

The complete lack of cleavage of the P1′ tryptophan mutant and the two mast cell chymase sites, even after prolonged cleavage, also shows the very high specificity displayed by human thrombin. This is in marked contrast to many other serine proteases, for example the mast cell chymases, which after prolonged cleavage shows activity towards a relatively broad range of substrates [29], [30], [47].

A relatively detailed picture of all the eight positions that may contribute to the substrate specificity of human thrombin has now been obtained by the phage display analysis in combination with the recombinant substrates. These results may also promote the identification of novel substrates, for example by contributing consensus motifs and individual cleavage-susceptible sequences for database searches. Our ProSite search with the refined consensus P-R-[AGST]-[not DE]-R has lead to the identification of 73 potential novel targets (Tables 3, 4, 5, and 6). These group in the fields of cell adhesion, the nervous system, development/differentiation and circulatory homeostasis. However, when evaluating these new potential substrates we need to keep in mind the possibility that numerous potential targets may be missed, due to that most of the *in vivo* sites that so far have been identified are not consensus sites. However, a very broad screening gives too many potential sites to be able to handle in this type of analysis.

Thrombin has previously been shown to digest numerous ECM components, such as nidogen, fibronectin, laminin and type V collagen [48], [49], [50]. The potential novel substrate integrin αV is therefore especially interesting, because it is a receptor for several previously known thrombin substrates, including prothrombin, fibronectin and laminin. The digestion of these central ECM molecules by thrombin may have important medical implications, for example in preventing the metastatic crossing of the ECM by tumors, and in wound healing.

The idea that thrombin may play an important role in the development and function of the brain has previously been indicated from the fact that the protease is expressed in various brain regions, especially during development and in regions exhibiting plasticity [51]. Thrombin has also been implicated in pathologic brain conditions, including adverse processes following CNS injury [52], [53], [54], [55], [56], and may influence the direction of neurite outgrowth [57]. Indeed, several novel candidate substrates have neurotropic functions, such as Roundabout homologs 1, -2 and –3 and persephin. Although the described target for many processes in the nervous system by thrombin is PAR-1 [5], [53], [56], [58], [59], [60], alternative targets may very well exist.

In summary, the use of substrate phage display technology in combination with the newly developed recombinant substrates has made it possible to determine the substrate recognition profile of the active site of thrombin, from position P4 to P4′, completely and simultaneously. This study has also resulted in a very detailed picture concerning kinetics, in relative terms, on the contribution by individual residues on cleavage specificity. The combination of these two techniques has made it possible to study the specificity of the catalytic site excluding interactions depending on exosite interactions. The obtained profile conforms very well with previous studies, and adds important kinetic parameters to these results with substrates having not only aa either N or C terminal of the cleavage site but the entire eight aa acids of the extended specificity. One very interesting finding is that most natural substrates are not optimal substrates for thrombin, which indicates that cleavage of such sites in the absence of strong exosite interactions, may lead to a too efficient cleavage, and this may cause excessive coagulation and a risk of unwanted thrombosis. Exosite interactions may here facilitate the interaction between enzyme and substrate and increase the kinetics and also substrate specificity in cleavage. In addition, the use of the consensus site to screen the human proteome has resulted in the identification of a panel of 73 potentially novel substrates for thrombin, some of them may prove to be important targets for this multi-facetted enzyme.

## Materials and Methods

### Thrombin

Lyophilized powder of human plasma thrombin (SIGMA T-6884) was diluted in double-distilled water to a concentration of 0.2 NIH units/µl. One U or 0.2 U of diluted thrombin were used in two separate phage display analyses.

### Analysis of thrombin's extended recognition sequence by substrate phage display

The cleavage specificity of thrombin was investigated with a T7 phage-displayed peptide library containing approximately 5×10^7^ individual nonamers as previously described [21], [28], [33], [47]. In this library, randomized nonamers are inserted in the carboxy-terminus of T7 capsid protein 10A, followed by a six-histidine tag (His_6_-tag) for purification. The constant region at the amino-terminal flank of the peptides consists of the aa proline-glycine-glycine, breaking any secondary structures imposed by the capsid protein. In brief, phages are anchored to nickel nitrilotriacetic acid (Ni-NTA) beads via the His_6_-tags before the first protease treatment. The protease is added and allowed to react over night, releasing phages displaying cleavage-susceptible 9-mers from the beads. Samples are centrifuged and cleaved phages are collected in the supernatant. These phages are amplified in the *E. coli* strain BLT5615 and enter the next selection round (biopanning). After five biopannings, enriched cleavage-susceptible phages are sequenced.

For the analysis of thrombin, an aliquot of approximately 10^9^ plaque-forming units was immobilized on 100 µl Ni-NTA agarose beads and incubated 1 hr with gentle rotation at 4°C. Unbound phages were removed by ten washes with 1.5 ml 1 M NaCl, 0.1% Tween-20 in PBS, pH7.2, and two subsequent washes with 1.5 ml PBS. The beads were resuspended in 1 ml PBS. One U or 0.2 U of thrombin were added and control samples with PBS instead of protease were run in parallel. Zero point two U/ml corresponds to a thrombin concentration of approximately 1.5 nM. Note the 137 mM sodium concentration in PBS implies that most of the thrombin is expected to be in the “fast” form [61]. Digestion and mock-digestion proceeded over night at room temperature under gentle rotation. Samples were centrifuged briefly on a tabletop centrifuge, pelleting the Ni-NTA beads. A control elution of the phages remaining bound to the Ni-NTA beads, using 100 µl 100 mM imidazole, concluded that at least 1×10^8^ phages were attached to the matrix in each selection round. Cleavage-susceptible phages were recovered, amplified and selected in five rounds as described earlier [21], [28], [33].

Fifty plaques were then arbitrarily isolated from Luria broth (LB) ampicillin (amp) plates representing biopannings with 1 U and 0.2 U of thrombin. Each plaque was dissolved in 100 µl phage extraction buffer (100 mM NaCl and 6 mM MgSO_4_ in 20 mM Tris-HCl, pH 8.0) and shaken vigorously for 30 min. Phage DNA from the variable region in the capsid 10A gene was amplified by PCR with vector-specific primers. PCR fragments were purified with the Omega-BioTech's E.Z.N.A™ micro elute kit (Omega Biotech, Vancouver Island, Canada). Purified PCR fragments were sequenced on an ABI PRISM® 3700 DNA Analyzer. Nineteen and 18 individual inserts coding for cleavage-susceptible peptides were sequenced from plaques representing selection with 1 U or 0.2 U of thrombin, respectively. One sequence containing a stop mutation (from selection with 1 U thrombin) and one sequence file of bad quality (from selection with 0.2 U of thrombin) were discarded.

### Alignment of sequenced phage inserts

Phage insert sequences were aligned with the program Consense (Anders Kaplan and Maike Gallwitz, unpublished). Settings were chosen to retrieve patterns of at least four aa. Approximately functional equivalent aa were grouped as follows: aromatic aa (phenylalanine, tyrosine, tryptophan); negatively charged aa (aspartate, glutamate), positively charged aa (arginine, lysine, histidine), small aliphatic aa (glycine, alanine), larger aliphatic aa (valine, leucine, isoleucine), others (proline, serine, threonine, cysteine, methionine, glutamine, asparagine).

### Generation of recombinant substrates for the analysis of the cleavage specificity

A new type of substrate was developed to verify the results obtained from the phage display analysis. Two copies of the *E. coli* thioredoxin gene were inserted in tandem into the pET21 vector for bacterial expression (Fig. 3A). In the C-terminal end, a His_6_- tag was inserted for purification on Ni-NTA agarose beads (Qiagen, GmbH, Hilden, Germany). In the linker region, between the two thioredoxin molecules, the different substrate sequences were inserted by ligating double stranded oligonucleotides into two unique restriction sites, one *BamHI* and one *SalI* site (Fig. 3A). The sequences of the individual clones were verified after cloning by sequencing of both DNA strains. The plasmids were then transformed into the *E.coli* Rosetta gami strain for protein expression (Novagen, Merck, Darmstadt, Germany). A 10 ml overnight culture of the bacteria harbouring the plasmid was diluted 10 times in LB Amp and grown at 37°C for 1–2 hr until the OD_600_ reached 0.5. Isopropyl β-D-1-thiogalactopyranoside (IPTG) was then added to a final concentration of 1 mM. The culture was grown at 37°C for an additional 3 hr under vigorous shaking, after which the bacteria were pelleted by centrifugation at 3500 rpm for 12 min. The pellet was washed once with 25 ml PBS and 0.05% Tween 20. The pellet was then dissolved in 2 ml PBS and sonicated 6×30 seconds to open the cells. The lysate was centrifuged at 13000 rpm for 10 min and the supernatant was transferred to a new tube. Five hundred µl of Ni-NTA slurry (50∶50) (Qiagen, Hilden, Germany) was added and the sample was allowed to slowly rotate for 45 min at RT. The sample was transferred to a 2 ml column allowing the supernatant to slowly pass through the filter leaving the Ni-NTA beads with the bound protein in the column. The column was washed four times with 1 ml of washing buffer (PBS, 0.05% Tween, 10 mM Imidazole, 1 M NaCl). Elution of the protein was achieved by adding 150 µl elution buffer (PBS, 0.05% Tween 20, 100 mM Imidazole) followed by five 300 µl fractions of the elution buffer. Each fraction was collected individually. Ten µl from each of the eluted fractions was mixed with 1 volume of 2× sample buffer and 1 µl β-mercapto-ethanol and subsequently heated for 3 min at 80°C. The samples were analyzed on 4–12% pre cast SDS bis-tris PAGE gels (Invitrogen, Carlsbad, CA, USA) and the fractions that contained the most protein were pooled. The protein concentration of the combined fractions was determined using the Bio-Rad DC Protein assay (Bio-Rad Laboratories Hercules, CA USA). Approximately 60 µg of recombinant protein was added to each 120 µl cleavage reaction (in PBS). Twenty µl from this tube were removed before adding the enzyme, the 0 minute time point. The active enzyme was then added and the reaction was kept at room temperature during the entire experiment. Twenty µl samples were removed at the indicated time points (15 min, 30 min, 45 min, 60 min and 150 min) and the reactions were stopped with the addition of one volume of 2× sample buffer. One µl β-mercapto-ethanol was then added to each sample followed by heating for 3 min at 80°C. Twenty µl from each of these samples was analyzed on 4–12% pre-cast SDS bis tris PAGE gels (Invitrogen, Carlsbad, CA, USA). The gels were stained over night in colloidal Coomassie staining solution and de-stained for several hours according to previously described procedures [62]. The intensity of the individual bands on the gel was determined from scanned high-resolution pictures by densitometric scanning of the gels and the program ImageJ (rsb.info.nih.gov/nih-image/). In order to obtain good estimates of the differences in activity towards different substrates different concentrations of the enzyme were used in several individual experiments. The combined results from these different gels were then used to get an accurate estimate of the difference in activity against the various substrates.

### PROSITE scan for the Phage Display consensus motif

The Swiss-Prot, TrEMBL and PDB databases were searched by a PROSITE Pattern Scan (http://www.expasy.ch/tools/scanprosite/) for human (*Homo sapiens*) proteins holding the consensus motif derived from sequenced phage inserts, P-R-[AGST]-[not DE]-R. No description filter was chosen, at most one character was allowed to match a conserved position in the pattern, and the match mode was set to “greedy, overlap, no includes”. Protein hits were assessed individually for the localization of the motif, expression pattern and presumable function. A number of hypothetical or poorly characterized proteins were not further assessed.
